# Characterization of radiographers’ mammography practice in five European countries: a pilot study

**DOI:** 10.1186/s13244-019-0711-0

**Published:** 2019-03-13

**Authors:** Nicole Richli Meystre, Anja Henner, Cláudia Sà dos Reis, Bergliot Strøm, José A. Pires Jorge, Tiina Kukkes, Eija Metsälä

**Affiliations:** 1 School of Health Sciences, University of Applied Sciences and Arts Lausanne, Western Switzerland, Lausanne, Switzerland; 2 Oulu University of Applied Sciences, Oulu, Finland; 3 Lisbon School of Health Technology, Curtin University, Medical Radiation Sciences, Perth, Western Australia; 4 Western Norway University of Applied Sciences, Bergen, Norway; 5 Tartu Health Care College, Tartu, Estonia; 6 Helsinki Metropolia University of Applied Sciences, Helsinki, Finland

**Keywords:** Mammography, Radiographer, Quality control, Professional practice, Challenges

## Abstract

**Objectives:**

This pilot study aimed to characterize and compare radiographers’ mammography practice, including quality control and continuous professional development in five European countries.

**Methods:**

Online survey was performed to collect data regarding participants’ profile, institution’s profile, mammography practice, quality control and continuous professional development. The questionnaire was sent to clinical radiographers working in Estonia, Finland, Norway, Portugal and Switzerland. Descriptive statistical and subgroup analyzes were performed.

**Results:**

The amount of returned questionnaires was 140. Most respondents were female (92%), having radiography bachelor. The majority (89%) of radiographers was working with full-field digital mammography. The majority (97%) of mammography images were acquired using AEC, and half of the radiographers were using dose saving programmes suggested by the manufacturers. The most typical (50%) compression force ranged from 8 to 11 kg. Part of the radiographers (44%) did not know if their practice followed specific guidelines. The most challenging tasks in mammography identified by radiographers were patient positioning (86%), coping with pain (88%), managing anxiety (83%) and imaging breast implants (71%). The majority (88%) of the respondents undertook continuous professional development activities.

**Conclusions:**

The mammography practice varies across the five countries. We found country-specific traits related to mammography image acquisition, patient-centered care and quality management procedures. The lack of evidence-based knowledge suggests the importance of well-designed studies on these topics. The variability found in this pilot study encourages radiographers to question their own practice and teachers to review and revise the training programmes. Validation in larger studies including more countries is needed.

## Key points

- Full-field digital mammography (FFDM) is the most commonly used modality.
- Quality control is performed by radiographers.
- Various guidelines are in use across the countries.
- Challenges in mammography involve positioning, coping with pain and imaging of breast implants.
- There is a need for further education and more research in mammography.

## Introduction

Variation in medical practice is common. Despite the body of theoretical knowledge shared by the members of a healthcare profession, equipment available, personal experience, enthusiasm for certain procedures, constraints and social influence can promote and explain this variability [1, 2]. However, variation may also suggest an inappropriate service chain or a risk of harm to the patient. Practical guidelines and performance indicators are supposed to limit the variability and hence increase the quality and personalized care [3–5]. This also applies to mammography where high-quality mammograms are crucial for the success of breast pathology detection and consequently for the success of breast cancer screening programmes [6–11].

Radiographers have a central role in the achievement of high-quality mammograms as they are responsible for quality control procedures, patient care, breast positioning and compression [12, 13]. Image quality monitoring showed that quality criteria of the guidelines were not satisfactorily met in 3 to 50% of the mammograms [14–16]. Incorrect positioning was the most frequently reported reason for failing. It was assumed that there exists a wide range of practice traits, depending on the country but also on individual preferences. The assumption is supported by several reasons: radiographer training programmes and continuous professional development (CPD) vary from one European country to another [17]. Implementation of new modalities may induce variation in practice as new scientific knowledge and professional skills need to be developed while practitioners are already working with the digital technologies available. Mammography is performed in two different contexts namely population-based breast cancer screening programme and traditional clinical setting. Finally, several mammography guidelines are available side by side, showing slight differences in recommendations related to quality assurance procedures and quality control tests [18, 19] as well as to training activities for the concerned healthcare staff.

Transition from screen-film mammography (SFM) to full-field digital mammography (FFDM) has had an impact on the organization of breast cancer screening programmes. Furthermore, the digital environment offers workflow efficiency that may lead to an increasing number of women scheduled per hour [20]. Temporarily increased recall rates have been observed which might suggest a digital mammography learning curve for radiologists [21, 22]. The wide implementation of FFDM has also had an impact on radiographers’ daily practice with upgraded quality control procedures, extended possibilities of dose optimization, post processing and new forms of artefact recognition and management [12]. All manufacturers offer at least two different automatic exposure control (AEC) modes, each one associated with a characteristic calibration curve that promotes either the contrast to noise ratio or a lower mean glandular dose (MGD). Individual preferences as well as in-house protocols may influence the choice between AEC modes developed by the manufacturers [23]. A recent study showed that the individual radiographer factor appeared to influence a woman’s decision to re-attend to her next screening round [24]. Nevertheless, only few studies have explored variation in mammography practice among radiographers with the exception of specific issues like compression force applied to the breast during mammogram acquisition [16, 25] and clinical quality assessment procedures [26].

The aim of this pilot study was to characterize radiographer-related mammography practice, including quality control (QC) and continuous professional development (CPD) in five European countries. The first objective was to identify the areas where mammography practice is harmonized vs. those areas where a variation is observed. The second objective was to identify the specific knowledge that needs to be disseminated among radiographers and students in order to support them to achieve high image quality in mammography.

## Materials and methods

The design of this pilot study was a cross-sectional survey based on an online questionnaire, involving clinical radiographers in Estonia, Finland, Norway, Portugal and Switzerland. These five countries are all included in the project called “Education and training in early detection of breast cancer for health care professionals” (EBreast project^1^). Our questionnaire was designed to gain data about practice and habits of radiographers and clinical tutors when performing mammography, quality control, practice of continuous professional development and perceived challenges in order to identify the areas where radiographers and students need evidence-based knowledge.

### Sample

The web link to the online questionnaire was sent to mammography radiographers either by the national radiographers’ society (Finland and Switzerland) or by the educational institutions involved in the EBreast project (Estonia, Norway and Portugal). An information sheet, sent with the web link, invited mammography radiographers to participate in the survey.

In order to facilitate the participation, the questionnaire was translated (one-way translation) into the Finnish, French, German and Portuguese languages. A recall to participate was sent 2 weeks before the end of the data collection.

Ethical rules were applied according to the policy of the participating countries. The participants were informed that their participation was voluntary and the data was collected and treated confidentially.

### Questionnaire design

The 45-item questionnaire was divided into four sections. The first one was about demographic information concerning the participants, including age, gender, country of residence, academic background and the number of years of professional experience in radiography and in mammography. This information allowed the characterization of the profile of the respondents. The second section was about the activities and workload, including available equipment and the number of mammograms per shift. The third section covered mammography image acquisition, technique, exposure parameters, breast compression, and quality assessment, including the preferred guidelines and quality control procedures. The fourth section surveyed training issues, including the perceived challenges and CPD. The questions were mostly closed-ended with the possibility to add comments. The answers were dichotomous, multiple choice or with a 5-point Likert scale.

Prior to the distribution, the questionnaires were tested in a sample of three radiographers from each participating country. Their suggestions were introduced in questionnaires to improve the tool.

### Analysis

Unanswered and improperly answered questions due to misunderstanding of the number of hours for a shift or the number of mammography exams performed during the shift were excluded from data. Because of this reason, the total number of respondents may vary for each question.

All analysis was performed by using the software package SPSS (version 21, IBM) with descriptive methods. The results were stratified by country, but because of the limited number of participants, the *p* value was not considered. The open-ended responses were grouped according to their subject.

## Results

The results describe and compare the mammography radiographers’ work environment, technical parameters applied, quality assurance procedures for equipment and for clinical mammography assessment, the guidelines in use and practice of continuous professional development.

One hundred forty questionnaires were returned. The estimated response rate was 21%. No precise response rate could be calculated for Estonia and Switzerland as there exists no exhaustive list of radiographers performing mammography for these countries [27]. The link to the questionnaire was sent to mammography departments, and in Switzerland, it was also available on social media. Therefore, the number of radiographers invited to participate in this pilot study could not be gathered afterwards and had to be estimated (Table 1).

**Table 1:** Number of questionnaires and response rate by country

Country	Number of questionnaires sent	Number of answers obtained	Response rate (%)	Comments
Finland	81	38	47	
Norway	47	10	21	
Portugal	33	19	58	
Estonia	25 (estimated)	8	50 (estimated)	Estimation takes into account that there exist five mammography centers in Estonia having an average of five radiographers performing mammography per centre.
Switzerland	500 (estimated)	66	13 (estimated)	Estimation is based on a report published by Swiss Society of Radiographers^1^
Total	686 (estimated)	141	21 (estimated)	

^1^ https://www.svmtra.ch/files/Dokumente/Verband/Projekte/120518wm_f_03_arbeitsmarktanalyse_schlussbericht.pdf

Our sample consisted of 6% Estonian (*n* = 8), 27% of Finnish (*n* = 38), 7% of Norwegian (*n* = 10), 13% of Portuguese (*n* = 18) and 47% of Swiss radiographers (*n* = 66).

### Participants’ background factors

Most respondents were female (92%). All age groups were represented (20 to > 60 years); 71% had at least a bachelor degree and 4% had a master’s degree. The mean of the years of experience in mammography clinical practice was 12 (ranging from 1 to 30 years). Respondents were working in various types of healthcare institutions, including public, private, public/private partnership institutions and university hospitals.

### Activities, equipment and workload related to mammography examinations

Diagnostic mammography in clinical setting was the main activity for 97% of the respondents, followed by screening mammography as a population-based programme (78%). Follow-up and intervention examinations were also performed by 47% and 90% of the respondents, respectively.

**Fig. 1:**
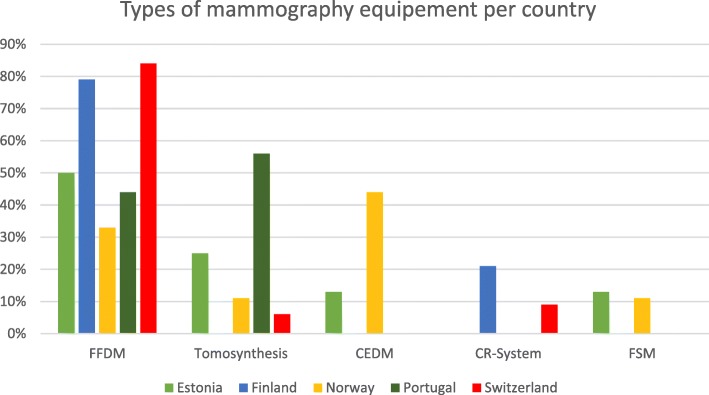
Types of mammography equipment used by participants per country

Full-field digital mammography (FFDM) was available in all participating countries, and it was the equipment most frequently used by responding radiographers (89%). Tomosynthesis (DBT) and contrast-enhanced digital mammography (CEDM) were available for 12% and 3% of the participants, respectively. Computed radiography (CR) system and screen-film mammography were available for 10% and 1%, respectively. The use of the CR-system was limited to two countries (Fig. 1).

Taking into account the manufacturers, the following equipment was in use: 36% General Electric (GE), 36% Siemens, 14% Hologic, 13% Planmed, 6% Philips and 5% Fuji. Some participants (8%) did not specify the manufacturer of their equipment. Seventeen percent of the participants used equipment of two or more manufacturers; this explains the sum of 118% of the equipment related to different manufacturers.

The mean time for one shift was 7 h (95% CI 6.7–7.3). On average, 21 mammography examinations (95% CI 17.4–23.7) were performed during a shift in the population-based screening programme while 12 mammography examinations (95% CI 10.6–13.3) were performed during the same time in the diagnostic setting. On average, radiographers had 23 min (95% CI 20–25 min) to perform a mammography exam in the screening programme and 56 min (95% CI 47–66 min) in the diagnostic setting (Table 2).

**Table 2:** Minutes per mammography examination in screening and in diagnostic settings

Country	Minutes per mammography examination in screening setting	Minutes per mammography examination in clinical setting
Estonia	17.4 (95% CI 9.76–25.03)	26.69 (95% CI 19.35–34.04)
Finland	23.2 (95% CI 18.25–28.17)	89.27 (95% CI 63.44–115.10)
Norway	14.03 (95% CI 6.28–21.78)	33.47 (95% CI 23.15–43.79)
Portugal	28.66 (95% CI −30.60-87.93)	28.88 (95% CI 16.65–41.11)
Switzerland	23.82 (95% CI 20.44–27.19)	39.23 (95% CI 31.38–40.08)
Mean	22.62 (95% CI 20.17–25.07)	56.13 (95% CI 46.75–65.51)

### Mammographic technique: AEC use, exposure parameters, breast compression, angulation for oblique views

*The automatic exposure control* (AEC) system was the most often used system (97%). However, the selection of the AEC mode varied. The manufacturer-specific dose saving mode was used by 50% of the radiographers. Half of the radiographers (55%) used several AEC modes. When asked what kind of anode/filter combination they would use for a mammography with 50:50 glandularity and 5.5 cm compressed breast thickness, 13% of the radiographers indicated an anode/filter combination that is not offered by the manufacturer of their mammography equipment. Some radiographers (6%) indicated that they were not sure about the anode/filter combination they used because of the automatic selection performed by the machine. The preferred anode/filter combination was Rh/Rh for GE (40%); W/Rh for Siemens (45%); Mo/Rh (33%) and W/Rh (19%) for Hologic, depending on the equipment in use; W/Rh for Fuji (67%) and Mo/Rh for Planmed (42%) (Table 3).

**Table 3:** Distribution of anode/filter combination per manufacturer which is “often” or “always” used by radiographers for a mammography with 50:50 glandularity and 5.5 cm compressed breast thickness ((%) *=* percentage)

Manufacturer	Mo/Mo	Mo/Rh	Rh/Rh	W/Rh	W/Ag	W/Al	Number of users
General Electric	14 (22)	17 (26)	26 (40)	8 (12)	–	–	65
Siemens	13 (26)	15 (29)	–	23 (45)	–	–	51
Hologic	5 (24)	7 (33)	–	4 (19)	1 (5)	4 (19)	21
Fuji	0 (0)	2 (33)	0 (0)	4 (67)	–	–	6
Philips	–	–	–	–	–	1 (100)	1
Planmed	3 (25)	5 (42)	–	0 (0)	4 (33)	–	12

The technique to compress the breast varied between countries. In Finland, only 3% of the respondents were applying minimal regulated compression force, while in Portugal, 67% of the respondents were following that strategy. Half of the respondents (50%) used compression force between 8 to 11 kg, while 26% of them preferred a higher compression force (from 11 to 15 kg). Compression force from 11 to 15 kg was the most used in Estonia (38%), Finland (53%) and Norway (68%). Compression force from 8 to 11 kg was the most used in Portugal (39%) and Switzerland (74%). Adapting compression force according to patient feedback was a strategy used by 47% of the respondents in order to know when to stop the breast compression. The use of this practice varied from 17% among Portuguese respondents to 87% of responding radiographers in Finland.

Selection of the equipment angle to perform the mediolateral oblique (MLO) projection varied: 39% of the respondents selected always a 45° angle, 32% selected always a 60° angle and 25% selected the angle according to the patient body habitus, considering the angle of the pectoral muscle. Differences between the countries were observed as follows: 38% of respondents in Estonia and 72% in Switzerland preferred a 45° angle while a 60° angle was preferred in the other countries.

### Quality assurance and quality control (QC) activities

Technical quality control (QC) was performed in the majority (99%) of the departments participating in this pilot study. Some of the respondents (3%) were not involved in the routine tests or did not do them (1%) because technical QC was not implemented. Daily and weekly technical QC tests were performed by 42% of the participating radiographers (Table 4). Different aspects were being routinely assessed such as detector stability, artefacts, image printing, CR-specific tests, monitor assessment and safety checks of the examination room and equipment. Differences in the frequency of quality control tests performed by radiographers were observed between countries (Table 5).

**Table 4:** Frequency of quality control tests performed by radiographers presented by country (*n* = frequency; (%) = percentage)

	Estonia	Finland	Norway	Portugal	Switzerland	Total
Detector stability test and flat-field phantom image						
Never	1 (13)	0	0	0	7 (11)	8 (6)
Daily	7 (87)	37 (97)	8 (89)	10 (56)	7 (11)	69 (50)
Weekly	0	0	1 (11)	2 (11)	49 (74)	37 (52)
Monthly	0	0	0	2 (11)	3 (4)	5 (4)
Quarterly or semi-annually	0	1 (3)	0	4 (22)	0	5 (4)
QC test object and full-field artefacts						
Never	4 (51)	0	6 (67)	2 (11)	5 (8)	17 (12)
Daily	2 (25)	24 (63)	2 (22)	1 (6)	0	29 (21)
Weekly	1 (12)	11 (29)	0	8 (44)	59 (90)	79 (57)
Monthly	0	1 (3)	0	3 (17)	1 (1)	5 (4)
Quarterly, semi-annually or annually	1 (12)	2 (5)	1 (11)	4 (22)	1 (1)	9 (6)
Artefacts						
Never	1 (12)	0	1 (11)	3 (18)	2 (3)	7 (5)
Daily	1 (12)	27 (71)	1 (11)	11 (62)	25 (38)	65 (47)
Weekly	5 (64)	6 (16)	6 (67)	1 (5)	38 (58)	56 (40)
Monthly	0	3 (8)	0	2 (10)	1 (1)	6 (4)
Quarterly, semi-annually or annually	1 (12)	2 (5)	1 (11)	1 (5)	0	5 (4)
Monitor QC						
Never	6 (76)	4 (11)	7 (78)	9 (50)	8 (12)	34 (25)
Daily	0	9 (24)	1 (11)	2 (11)	5 (8)	17 (12)
Weekly	1 (12)	13 (34)	0	4 (22)	44 (67)	62 (45)
Monthly	0	5 (13)	0	1 (6)	4 (6)	10 (7)
Quarterly, semi-annually or annually	1 (12)	7 (18)	1 (11)	2 (11)	5 (7)	16 (11)
Printer/laser printer						
Never	0	34 (90)	0	15 (83)	42 (64)	91 (66)
Daily	3 (37)	1 (3)	4 (44)	2 (11)	3 (4)	13 (9)
Weekly	4 (50)	0	5 (56)	0	11 (17)	20 (14)
Monthly	1 (13)	1 (3)	0	0	4 (6)	6 (4)
Quarterly, semi-annually or annually	0	2 (4)	0	1 (6)	6 (9)	9 (7)

**Table 5:** Frequency of safety checks of examination room and equipment performed by radiographers presented by country (*n* = frequency; (%) = percentage)

Safety and function checks of examination room and equipment	Estonia	Finland	Norway	Portugal	Switzerland	Total
Never	0	3 (8)	1 (11)	3 (17)	15 (23)	22 (16)
Daily	1 (12)	7 (18)	1 (11)	11 (61)	19 (29)	39 (28)
Weekly	6 (76)	7 (18)	6 (67)	1 (6)	11 (17)	31 (22)
Monthly	0	4 (11)	0	1 (6)	5 (8)	10 (7)
Quarterly, semi-annually or annually	1 (12)	17 (45)	1 (11)	2 (10)	16 (13)	27 (17)

Clinical quality control was implemented in the departments, and it was performed by 87% of the respondents. Country-specific differences were observed, ranging from 100% of respondents performing it in Estonia and Finland down to 44% in Portugal. Reject image analysis was routinely performed by 53% of the participants.

**Fig. 2:**
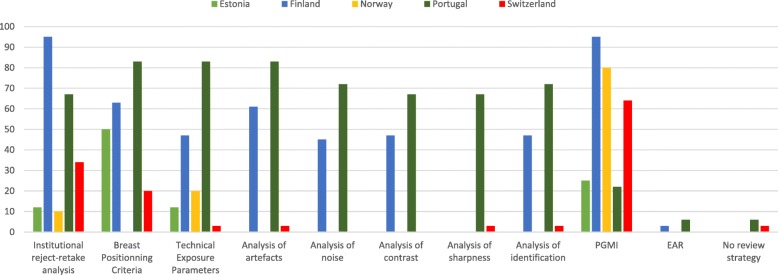
Criteria used by radiographers to assess the quality of clinical mammography images

The quality of the mammograms produced in the clinical setting was reviewed by 98% of the respondents. The four-level quality system, known as the PGMI system (perfect–good–moderately good–inadequate), was the mostly (66%) preferred one. This system includes quality criteria related to breast positioning, technical parameters, sharpness, artefacts and patient identification. By compiling the PGMI strategy with specific criteria, 87% of the respondents checked the positioning of the breast, 79% reviewed the technical parameters, 76% the artefacts and 75% the sharpness. Country-specific differences were observed for the review of technical parameters, artefacts and sharpness (Fig. 2).

### Guideline use related to technical performance of mammography

There was a variation between the participating countries in the use of recommendations related to technical performance. Twenty-five percent of the participants named international guidelines given by the EUREF (9%), the American College of Radiology (7%) and the IAEA (4%), while 23% named national guidelines and 5% local/departmental ones. Nearly half (44%) of the respondents could not identify the guidelines used in their unit. Similar results were found for diagnostic reference levels (DRLs). There were differences between the countries: in Estonia (50%), Finland (87%) and Norway (56%), the most commonly used DRLs were the national ones; in Switzerland, the local ones (39%) and in Portugal, the American DRLs were a reference for 44% of the respondents.

### Challenges in clinical practice perceived by radiographers

Irrespective of the country, the majority of respondents considered positioning (86%), coping with pain (88%) and anxiety (83%) as well as positioning of the breast with implants (71%) as the most challenging tasks. Some respondents also found patients with physical disability, also overweight or allophone patients, as challenging in clinical practice. To perform biopsies and tomosynthesis were also mentioned as challenging clinical tasks.

### CPD practices and requirements

Irrespective of the country, the majority (88%) of the respondents participated in continuous professional development (CPD) regarding mammography. However, 62% of them had less than 10 h of CPD per year for updating their knowledge in this topic. Congresses (70%) and conferences (41%) were the most common sessions attended by participating radiographers to update the knowledge. Some respondents from Finland and Switzerland also highlighted the importance of courses organized by the national radiographers’ society. Most respondents (80%) reported the need for additional education in mammography. Most preferred topics for further education were new imaging techniques, pathology, image quality analysis and optimization in mammography examinations.

### Similar practice traits versus various practice traits

Various practice traits have been observed in five different areas including referral guidelines, choice of technical parameters and QC protocols (Table 6).

**Table 6:** Similar practice traits versus various practice traits

Traits characterizing all practices	Traits characterizing some practices
Majority of female radiographers	
FFDM was implemented in all participating countries	The use of CR-system and to lesser extent SFM in two countries The use of tomosynthesis in four countries
More time was allocated for diagnostic mammography than for screening mammography; 20-min time was allocated for a two-view screening mammography	
AEC widely used	Various AEC modes are used Use of different anode and X-ray beam filter material
	Application of compression force
QC of the equipment is performed QC in clinical mammography is performed	Frequency of equipment QC Reject-retake analyzes implemented in some departments Various criteria for QC of clinical mammography
	Various Referral guidelines

## Discussion

Achieving and maintaining high image quality is multifactorial. This pilot study provided an opportunity for an overview of radiographers’ profile, mammography equipment and clinical practice regarding mammography in five European countries.

Radiographers working in mammography were mainly females, aged between 20 and 59 years of age, with at least BSc level education and with further education and training in mammography acquired in congresses and conferences. These results are in line with other studies [28, 29] that reveal female radiographers as the preference to perform mammography since patients may feel uncomfortable when assisted by male radiographers.

A time period per mammography exam was shorter in the screening programme than in the clinical setting, 23 and 56 min respectively. This difference is due to a wide range of additional views and follow-up examinations performed in the clinical setting. The mean time per mammography exam in the clinical setting ranged from 27 to 89 min. Depending on the department, ultrasound exams and/or biopsies were performed in the mammography room. The mean time per mammography exam in the screening programme ranged from 8 to 40 min. The patient schedule in mammography was challenging for 44% of the participants. To our knowledge, the adequate time period for mammography exam has not been studied either in relation to radiographer and patient satisfaction or to image quality.

FFDM systems were installed in the majority of the departments. However, CR and to a lesser extent SFM systems were still in use in some departments. This variety can impact the technique and consequently the mean glandular dose and image quality [30]. The cancer detection rate is also affected by the detector type, and the use of CR is controversial [31–33].

Although radiographers can choose between different AEC modes, 45% of them always use the same AEC mode. Dose saving AEC modes were used by 50% of the participants. Using this mode is especially important in a population-based breast cancer screening programme where mainly healthy women are exposed to ionizing radiation. In our sample, 20% of the participants did not practice mammography in a screening programme. This could partially explain variation in the use of dose saving AEC mode. Different anode/filter material and kV selection have an impact on the energy of the produced radiation beams. Therefore, the selection of exposure parameters has an impact on the absorbed radiation dose as well as on the image quality. This pilot study did not explore whether the choice of the AEC mode is due to personal preferences or to the in-house protocols. However, it suggests that 13% of the radiographers do not precisely know about the anode/filter combination they use when performing a mammogram. We can assume that they delegate optimization of parameters to their equipment. These results are in line with other studies which found that radiographers choose the mode according to the manufacturer’s recommendations [34] or may be unfamiliar with the characteristics of different modes [23]. In most cases, the AEC of the FFDM systems successfully identifies appropriate exposure parameters; nevertheless, previous studies found room for improvement [35–38]. This suggests how important it is for radiographers to know about the forces and limits of the AEC modes they use. For example, for a mammography with 50:50 glandularity and 5.5 cm compressed breast thickness, Siemens users prefer the anode/filter combination W/Rh (40%). Depending on the equipment, Hologic users prefer the anode/filter combination Mo/Rh (33%) or W/Rh (19%). These results are in line with the findings demonstrated by Williams et al. [36]. W/Al combination was also mentioned by 19%. As this combination is only used for DBT, suggesting that DBT is now considered like a standard mammography by some radiographers. In this pilot study, 46% of GE users prefer the anode/filter combination Rh/Rh. This is in opposition to Williams et al. [36] who found that Mo/Rh outperformed Rh/Rh combination. Anode/filter combination Mo/Mo was never preferred by any manufacturer. This is in line with Dance et al. who found that this combination was suitable for SFM but an alternative spectrum was preferable for breasts thicker than 2 cm [39]. In SFM, the main effect of using a higher X-ray energy is to reduce the patient dose but the consequence is a loss in image contrast. For FFDM, the optimization process is different because this technology offers a wider dynamic range and a linear relationship between dose and signal intensity [30, 37]. The kV range has not been explored in this pilot study. Radiographers should be aware that different AEC modes have an impact on the dose and image quality. In our opinion, interprofessional collaboration that brings together radiographers, medical physicists and radiologists is needed to be sure that the best combination of anode/filter material associated to kV is applied to every woman. The principles of digital mammography, the impact of AEC mode in mammography and the manufacturers’ specific modes should be taught in the radiographer curriculum and developed later on in mammography CPD courses to radiographers. It is important to increase radiographers’ awareness in order to optimize technical parameters.

The breast compression technique can also affect both the dose and image quality besides the patient experience and pain/discomfort. For most patients, pain is related to the application of compression [40] and that has been pointed out as a crucial factor affecting the participation in breast cancer screening programmes [41, 42]. A recent study [43] showed that radiographers are aware that pain is evitable or at least might be reduced, meaning radiographers have an important role to play during the exam since they can adapt practice to provide a better experience to the patients. The majority (88%) of the radiographers participating in this pilot study highlighted that concern and referred to positioning as a very challenging task, especially when the exam is painful for the patient or when the patient is anxious. Nightingale et al. [44] discussed the importance of patients’ verbal and non-verbal feedback for compression adaptation. However, only 47% of the participants were applying compression force according to patient feedback. That can explain the variability in the mean compression force applied by the participants in this pilot study, ranging from less than 8 to 15 kg, and might be due to the absence of or differences in the recommendations regarding compression force levels [19]. Branderhorst et al. [45] found a number of factors contributing to the variation in compression, including the pain threshold of the woman, the radiographer’s sensitivity to pain expression, the uncertainty or inaccuracy in estimating the pressure on the breast and the radiographer’s opinion of what is a good compression. Some authors [46–48] suggest the existence of local compression culture related to the radiology department. Recent research results about the adverse effects of excessive compression force on the participation rate of breast cancer screening and on image quality need to be taught in the radiographer curriculum. Communication and social skills should be developed during mammography CPD courses for radiographers in order to increase their awareness related to appropriate compression of the breast.

The practice of angle selection during breast positioning for MLO view acquisition varied among our participants. Only 25% of respondents were selecting the angle according to the patient body habitus, considering the angle of the pectoral muscle. The majority of participants always selected only one angle (45° or 60°), irrespective of patient body habitus. That may lead to a suboptimal positioning, and the incorrect angle selection can result in excessive compression force and pain in the chest wall/axilla. This may cause unnecessary discomfort to the patient and also result in inadequate compression of the breast [49]. Evidence-based knowledge on this issue would support radiographers to apply the best angle.

QC tests are different for each system, requiring adjustments in the procedures affecting radiographers’ activities and therefore the harmonization of practice across Europe [17–19, 50]. Technical QC seems to be well implemented, and 96% of the respondents were familiar with the main tests. The variability was mostly related to the frequency of use of the tests. Variation in the frequency of test performance can be related to the multiplicity of the guidelines referred to by radiographers.

Clinical QC is implemented in the departments of 87% of the participants. Reject/repeat analysis was performed by half of the respondents. The reject/repeat analysis is an important part of QC [8, 9, 35, 50] because the reasons for repeated mammograms can be related to blurring, artefacts, equipment failure, improper exposure but mainly due to the inadequate breast positioning and radiographers’ performance [14, 16, 51–53]. The identification of retake causes as a quality measure can facilitate the implementation of corrective and preventive actions, assuring that mammographic images are produced in compliance with the ALARA principle [8, 9].

The assessment of mammographic image quality is a challenging task that does not only mean checking the positioning based on the criteria but also checking for blurring and sharpness. Hogg et al. [54] stated that blurred mammograms are the main cause of repeats. These authors assume that the increasing frequency of blurring observed with the implementation of FFDM is not due to a new phenomenon but it is related to the higher contrast resolution. Apart from the differences in the acquisition system, the visualization system also plays a role in the identification of blurring, mainly when high-resolution monitors are used. Ma et al. [55] showed that the technical recall rate for blurring was higher when the quality review was performed on 2.3-megapixel monitors than on a 5-megapixel monitor. In this pilot study, radiographers did not mention assessment of sharpness as a challenge, maybe because they were not yet aware of the impact and difficulty of identifying blurred mammograms in the clinical setting. The importance of quality criteria related to sharpness, spatial resolution and absence of artefacts in addition to positioning for quality assessment should be taught in the radiographer curriculum. Examples of blurred mammograms could be integrated into image quality reviews that could be discussed during the mammography CPD courses for radiographers.

National guidelines have been elaborated and were referred to by 23% of the participants, showing that international guidelines do not fit to all screening conditions across Europe. In this pilot study, it was not possible to find out whether the national guidelines are inspired by international guidelines. The use of guidelines and DRLs varied not only across the five countries but also among the participants from one and the same country. Moreover, 44% of the participants could not identify the guidelines used in their department and some participants even indicated to refer to national DRLs in counties where no national DRLs exist. This may indicate that some radiographers adapt their practice to the habits of their department, without questioning the origin of the procedures. This could also indicate the need to promote the importance of evidence-based practice among radiographers.

Most of the participants (80%) expressed their need for more training. Van Landsveld-Verhoeven et al. [56] found that despite a mandatory CPD activity every 3 years, there was a decrease in the positioning quality in the Netherlands compared to the initial positioning quality. Those results highlight the need for setting individual targets, continuous monitoring and, when necessary, providing more frequent training to keep mammographic positioning skills up to date.

Limitations of this pilot study are related to a small number of participating countries and the response rate, limiting the generalization of results. Moreover, it was not possible to calculate the response rate in Switzerland and Estonia. The translation of questionnaires into different languages may have had an impact on the responses. The data analysis was performed without weighting the sample according to the number of participants per country.

In conclusion, this is the first cross-sectional pilot study exploring the variability of mammography practice among radiographers in several European countries. Beyond individual variability, we found country-specific traits related to mammography production, patient-centred care and quality management procedures. This highlights the need for a large and well-designed European survey with sufficient statistic power on this subject. The majority of the radiographers shared the need for more training. The lack of evidence-based knowledge, especially related to the clinical quality criteria of images and optimal breast compression, suggests the importance to develop research projects on these topics. The variability found in this pilot study could encourage radiographers to question their own practice and teachers to review and revise the training programmes.

## Footnotes

1 The EBreast project aimed to produce interprofessional mixed mode Open Access e-learning packages to increase the competences of early breast cancer detection and diagnostic procedures among health care professionals, students and teachers in the field, following the principles of interprofessional education. The consortium included higher education institutions, a hospital and a radiation protection authority from five European countries (Estonia, Finland, Norway, Portugal and Switzerland). However, to produce the e-learning packages, studies were conducted to collect data that allows the identification of the relevant content for the professionals involved in education, training and clinical practice regarding breast cancer diagnosis.
